# Editorial: Molecular and Metabolic Mechanisms Associated with Fleshy Fruit Quality

**DOI:** 10.3389/fpls.2017.01236

**Published:** 2017-07-13

**Authors:** Ana M. Fortes, Antonio Granell, Mario Pezzotti, Mondher Bouzayen

**Affiliations:** ^1^Faculdade de Ciências de Lisboa, BioISI, Universidade de Lisboa Lisboa, Portugal; ^2^Instituto de Biología Molecular y Celular de Plantas (CSIC-UPV) Valencia, Spain; ^3^Department of Biotechnology, University of Verona Verona, Italy; ^4^Laboratory of Genomics and Biotechnology of Fruit, INRA, University of Toulouse Toulouse, France

**Keywords:** breeding, fruit ripening, fruit quality, grapevine, molecular mechanisms, metabolic profiling, tomato

## Introduction

Fleshy fruits constitute a commercially important and nutritionally indispensable food commodity. In 2014, the total production of tomatoes and grapes worldwide was 170,750,767 and 74,499,859 tones, respectively (FAOSTAT).

This issue covers the most recent advances in our understanding of different aspects of fleshy fruit biology. In fact, fruit development and ripening involves several processes that were addressed in this issue namely accumulation of bioactive compounds (Calafiore et al.; Docimo et al.; Karppinen et al.; Rigano et al.) and modification of components that affect nutritional quality (Baldina et al.; Barrantes et al.; Dai et al.; Karppinen et al.; Rambla et al.) as well as modifications in texture triggered by cell wall changes and increased susceptibility to pathogens (Alkan and Fortes; Hocking et al.; Prusky et al.). The reprogramming of fruit development and ripening involves several transcription factors (Arhondakis et al.; Docimo et al.; Grimplet et al.; Li et al.), hormones (Tijero et al.; Alkan and Fortes; Karppinen et al.), nitric oxide (Bodanapu et al.), light signaling (Llorente et al.), calcium (Hocking et al.), small ncRNAs (Paim Pinto et al.), and epigenetic regulation (Gallusci et al.). Furthermore, environmental cues (Joubert et al.; Karppinen et al.; Paim Pinto et al.; Ripoll et al.; Dal Santo et al.), cultivation practices (Conde et al.; Corso et al.; Habran et al.), and postharvest strategies (Tijero et al.; Li et al.; Liu et al.) were shown to have an impact in ripening properties and fruit quality traits. Finally, in the review by Gascuel et al. were addressed the available genetic resources for breeding purposes of commercially important commodities such as tomato and grape. Furthermore, the technologies that facilitate the identification of genes/alleles of interest within the natural or generated variability gene pool were explored.

## Regulation of fruit development and ripening

Fruit development and ripening involves hormonal regulation in which auxins, cytokinins, ethylene, and ABA play an important role among other hormones (Fortes et al., 2015). The involvement of ABA in promoting ripening and quality of climacteric and non-climacteric fleshy fruits was reported by several authors in this issue (Alkan and Fortes; Hocking et al.; Karppinen et al.; Li et al.; Tijero et al.).

Endogenous ABA was mentioned to be involved in the regulating of ripening of climacteric tomato fruit (Bodanapu et al.). Fruit ripening in the *sh*ort *r*oot (*shr*) mutant of tomato that hyperaccumulates nitric oxide was delayed compared with the wild type. Central carbon metabolism and endogenous phytohormones levels were affected in the *shr* fruits. The authors highlighted that a crosstalk among nitric oxide and auxin, ABA and ethylene may regulate ripening and that selective manipulation of nitric oxide levels during ripening may increase shelf life of tomato.

In sweet cherries, a non-climacteric fruit, Tijero et al. studied the role played by ABA during pre-harvest and post-harvest room temperature/cold treatments. Endogenous ABA concentrations positively influenced quality parameters (accumulation of anthocyanins and vitamin E) during pre-harvest but not during post-harvest. Cold treatment increased ABA levels and led to an inhibition of senescence. The authors concluded that endogenous ABA promotes fruit ripening on the tree, but delays over-ripening in detached fruits.

Transcription factors play an important role in the regulation of fruit ripening (Docimo et al.; Grimplet et al.; Li et al.). The GRAS and the NAP gene families were characterized in grape and peach, respectively (Grimplet et al.; Li et al.). Both families play an important role in plant growth and development. By comparing the information available for tomato and grapevine GRAS genes, Grimplet et al. identified candidate genes that might constitute conserved transcriptional regulators of both climacteric and non-climacteric fruit ripening and that deserve further functional analysis. Co-expression analysis of GRAS genes provided insights into the molecular networks related with development and stress responses involving these transcription factors. On the other hand, Li et al. identified peach *NAP* genes that are responsive to ABA post-harvest treatment and that may regulate peach ripening. ABA-treated fruits softened faster and released more ethylene resulting in a shorter maximum storage period. In accordance, the promoters of four fruit-specific NAP genes presented ABA-responsive elements. Moreover, Arhondakis et al. identified two transcription factors, a SlWRKY22-like and a SlER24 transcriptional activator which were shown to regulate modules by using the LeMoNe algorithm for the analysis of microarray datasets representing four stages of tomato ripening. The WRKY22-like module comprised a subgroup of six various calcium sensing transcripts. In agreement, the promoter of these genes contained a *cis- acting* element, the W-box, recognized by WRKY transcription factors that might be involved in their coordinated regulation of expression. This approach can be applied for the construction of general fruit ripening regulatory module networks in particular those involving transcription factors.

Manipulation of individual components of light perception and signaling networks in tomato (*Solanum lycopersicum*) affects the metabolism of ripening fruit (Llorente et al.). In this mini-review, the authors explored how molecular mechanisms originally devoted to respond to environmental light cues have been re-adapted during evolution to inform plants on fruit ripening progression. The spectral composition of the light filtered through the fruit pericarp can be transduced by phytochromes and phytochromes-interacting factors, respectively, to regulate gene expression and in turn modulate the production of carotenoids. This process involves recycling of light-signaling components to finely adjust pigmentation. This trait may have evolved as an advantageous trait. In fact, the ability to display a change in color when the fruit is ripe would attract more seed dispersers among early fleshy- fruited plants.

The effect of calcium on fruit ripening was documented in the review by Hocking et al. The authors reported on the major components that determine calcium supply and distribution in grape. Moreover, calcium-pectin cross-links are a key factor in determining pectin properties and therefore influence remodeling of fruit cell walls. In turn, this affects fruit mechanical properties (softening), water relations and pathogen susceptibility. Calcium is a secondary messenger during hormone signaling and therefore can influence ripening through interaction with hormones. The authors concluded that improved understanding of the calcium nutritional requirements of plants may aid in optimizing fruit quality as both calcium deficiency and toxicity can affect the productivity, characteristics and pathogen susceptibility of the fruit.

Recent strong evidence suggests that fruit ripening is under not only genetic but also epigenetic regulation (Gallusci et al.). In this review, it was described how post-translational modifications of histones influence chromatin organization and contribute to the epigenetic regulation of gene expression during fruit ripening. They further explored the impact of variation in DNA methylation levels on the expression of ripening-related genes. In tomato and probably in other species such as grape (Fortes and Gallusci, 2017) the process of fruit ripening requires active DNA demethylation (Liu et al., 2015). Changes in DNA methylation due to spontaneous mutations or genome duplications can lead to the generation of natural epialleles affecting fruit phenotypes. The authors concluded that epi-marks on gene promoter regions could be used for “fine tuning” of gene expression in breeding strategies and for crop improvement.

## Ripening associated processes and fruit quality

Karpinnen et al. focused on the mechanisms associated with the regulation of key secondary metabolites in *Vaccinium* berries. Bilberry is a very rich source of anthocyanins that start to accumulate with the onset of ripening. The variation in flavonoid profile during berry development is related to seed dispersal and defense responses, subjected to hormonal control and involves transcription factors namely from the R2R3 MYB family. Many berries also accumulate carotenoid derived volatile flavor compounds at ripening (Agudelo-Romero et al., 2013; Karppinen et al.). Furthermore, light conditions, temperature, altitude, and genotype X environment interactions affect the composition of secondary metabolites in fruits.

Docimo et al. reported on metabolite abundance, regulation of chlorogenic acid, and anthocyanin biosynthesis, and characterization of candidate chlorogenic acid biosynthetic genes in eggplant, a fruit known to accumulate health-promoting phenylpropanoids. Analysis of the promoters of the biosynthetic genes (*SmPAL1, SmHQT1, SmANS*, and *SmMyb1*) revealed the presence of several MYB regulatory elements. Furthermore, the authors also determined that deletion of the C-terminal region of SmMYB1 does not limit its capability to regulate chlorogenic acid accumulation, but impairs anthocyanin biosynthesis, proposing therefore a functional role of the C-terminal domain of this transcription factor.

Genomics resources were exploited in order to identify candidate genes underlying antioxidants content in tomato (Calafiore et al.). The authors used *Solanum pennellii* introgression lines harboring quantitative trait loci (QTL) that increase the content of these bioactive compounds in the fruit. The differential expression of six candidate genes associated to ascorbic acid and one with carotenoids' metabolism together with polymorphisms in the sequences of the wild and the cultivated alleles of these genes may account for increased content in these metabolites. In another work from the same group, two genotypes carrying loci from the same wild species were crossed and two genotypes carrying introgressions at the homozygous condition (DHOs) were shown to present increased antioxidants content, revealing a positive interaction between the two wild regions pyramided in DHO genotypes (Rigano et al.). In these genotypes, occurs a putative redirection of the phenylpropanoid flux toward the biosynthesis of phenolic acid glycosides. Gene mapping, transcriptional profiling and biochemical analyses suggested a central role of the 4-coumarate:CoA ligase in redirecting the phenylpropanoid pathways whereas Myb4 and bHLH transcription factors may regulate these pathways. This work highlighted that interaction effects between QTLs must be studied in order to design an efficient pyramiding strategy for increasing fruit nutritional quality.

On the other hand, Barrantes et al. evaluated the breeding potential of introgression lines from the *Solanum pimpinellifolium* accession TO-937 into the genetic background of the “Moneymaker.” The authors identified using a genomic library chromosomal regions associated with both vegetative and fruit-related traits. QTLs were detected for fruit weight and organoleptic traits whose stability across generations depended on the trait. Ballester et al. characterized fruits grown in two locations of a population of introgression lines derived from a cross between *Solanum lycopersicum* and the wild species *Solanum chmielewskii*. Robust metabolite QTLs were identified for content in flavonoids, phenylpropanoids and alkaloids. Furthermore, *chalcone isomerase 1* was identified as the key gene underlying the variation in quercetin- and kaempferol glycosides. Altogether, the results demonstrated that by combining genetic and genomic resources in tomato with bioinformatics tools and metabolomics, dissection of QTLs and mQTLs can be achieved in order to improve the nutritional value and attributes of tomato.

The need to improve organoleptic characteristics in fruits is driving attention toward wild relatives but also traditional fruit varieties. Baldina et al. studied the content of several metabolites in tomato landraces categorized into three broad fruit type classes. The round/elongate types showed a higher content in glycoalkaloids, whereas flattened types had higher levels of phenolic compounds and were rich in aminoacids in particular glutamate, a compound directly related to organoleptic quality. The positions of several SNPs markers showed correspondence with already described genomic regions and QTLs. This work indicated that the future detection of mQTLs for important metabolites will give valuable tools to improve traditional tomato varieties by assisted breeding.

Aroma compounds are key elements in fruit quality. Chen et al. explored the involvement of *Cucumis melo* alcohol dehydrogenases (ADHs) and alcohol acyl-transferase (AAT) in the formation of volatile organic compounds. Ethyl acetate and hexyl acetate (E,Z)-3,6-nonadien-1-ol were found to be the principle aroma compounds of two cultivars whereas (E, Z)-3,6-nonadien-1-ol was the most abundant volatile in the non-aromatic cultivar. Several *CmADH* genes were specifically expressed in ripe fruits and differences were noticed between aromatic and non-aromatic varieties; these genes may code for isoenzymes with different substrates preference. Total AAT activity but not ADH seems to regulate esters abundance. Finally, *CmADH3* and *CmADH12* were selected as putative candidates involved in the synthesis of aroma compounds of oriental melon.

In two grape varieties (one white and one red) the emission of volatile and non-volatile compounds during berry maturation was investigated (Rambla et al.). Early stages were characterized in both cultivars by higher levels of some apocarotenoids, terpenoids and several furans, while the final stages were characterized by the highest amounts of benzenoid phenylacetaldehyde, 2-phenylethanol, 3-methylbutanol, among others. The study also highlighted that different varieties may have different content in certain volatile precursors. By also monitoring the expression of genes putatively involved in the synthesis of these compounds, the authors explored gene-metabolite networks of volatile metabolism and establish candidate genes involved in aroma formation. Furthermore, correlation analysis showed a higher degree of overall correlation in precursor/volatile metabolite-metabolite levels in the white variety, highlighting the different mechanism occurring in white varieties to develop an enriched aroma bouquet.

One of the characteristics that makes the fruits attractive to human consumption is the soluble sugar concentration that depends on sugar import, sugar metabolism, and water dilution (Dai et al.).These authors performed an inter-species comparison in order to identify common and/or species specific modes of regulation in sugar accumulation. By using a mathematical framework for the analysis of 104 combinations of species (grape, peach, and tomato), genotypes, and growing conditions, the authors concluded that different regulation modes of soluble sugar concentration operate being either import-based, dilution-based, or shared. The distinct modes appear to be species-specific, but the intensity of the effect may depend on the genotype and management practices. These results provided novel insights into the drivers causing the inter-species variability in soluble sugar concentration in fleshy fruits.

Increased sugar concentration in ripe fruits leads to increased susceptibility toward pathogens as reviewed by Prusky et al. and Alkan and Fortes. During fruit ripening physiological shifts occur: cell wall remodeling, decrease in the amount of phytoanticipins and phytoalexins, decline of inducible host defense responses, cuticle biosynthesis and changes in the ambient host pH. These changes are regulated by a complex interplay of hormonal signals that involve ethylene, ABA, jasmonic acid, and salicylic acid, among others and they release the fungus from its quiescent state and promote a necrotrophic life style (Alkan and Fortes). Recent data suggests that carbon availability in the environment (sugar levels) is a key factor triggering the production and secretion of small pH-modulating molecules (ammonia, gluconic acid) that contribute to colonization by postharvest pathogens (Prusky et al.). These pathogens modulate the expression of genes contributing to pathogenicity according to environmental pH-inducing conditions. The authors emphasized that knowledge on the processes responsible for the onset of necrotrophic stage may lead to strategies aiming at enhancing fruit defense and decreasing fungal virulence that will result in increased quality of fruits.

## Impact of environmental cues, cultural practices, and postharvest strategies on fruit quality

Environmental factors such as water deficit may negatively impact fruit yield and quality. The effect of three successive cycles of moderate water deficit and recovery was analyzed during the plant reproductive period in parental accessions of Multi-Parent Advanced Generation Inter-Cross population which presents the largest allelic variability observed in tomato (Ripoll et al.). Independent responses were observed in the leaf and fruit whereas negative effects on fruit fresh weight were dependent on stress intensity. Fruit quality was improved under water deficit mainly through concentration effects. The authors concluded that responses to drought are strongly genotype-dependent as well as highly variable depending on the stage of development at the time water deficit was applied. However, repeated cycles of water deficit and recovery may be used to improve fruit taste if the full variability of genotypic responses and crop performance is explored considering developmental stages.

Joubert et al. concluded that grape berries employed carotenoids and the associated xanthophyll cycles to acclimate to UV exposure. The berry responses differed between high light and low light conditions, in particular when the berries are still photosynthetically active (green developmental stage). Furthermore, in the highlight environment, certain monoterpenes and norisoprenoids were decreased by UVB attenuation confirming that UVB exposure stimulates volatile organic compounds in exposed ripe berries. These volatile terpenes may play a role in stress sensing and signaling related with UVB radiation. This work extended the current understanding of light/UV impacts in grapes and their metabolic plasticity in response to this environmental cue providing valuable data for stress management and improvement of grape quality.

Phenotypic and metabolic plasticity were also addressed in a white berry variety grown at four sites presenting different pedoclimatic conditions (Dal Santo et al.). Several genes that control transcription, translation, transport, and carbohydrate metabolism showed different expression depending on the environmental conditions. An important conclusion from this work was that genes representing the phenylpropanoid/flavonoid pathway showed highly plastic responses to the environment mirroring the accumulation of the corresponding metabolites. Furthermore, phenotypic plasticity varies among cultivars and may depend on whether the berries are white or red, highlighting the importance of conducting these studies in order to understand how grape and wine characters are developed in different environments from the same genotype.

Not only the epigenome and the RNA transcriptome but also the small RNA transcriptome participates in the dynamic regulatory network occurring in genotype-environment interactions (Paim Pinto et al.). These authors studied grapevine berries at four developmental stages from two varieties growing in three different environments. The results indicated that the distribution of small RNA-producing loci is variable between the cultivars, with the two cultivars showing a completely different small RNA profile across environments. On the other hand, the profile of miRNA accumulation mainly depends on the developmental stage. Several known vvimiRNAs and novel vvimiRNA candidates presented an accumulation in the berries modulated by at least one of the variables studied. The *in silico* prediction of miRNA targets suggests their involvement in berry development and in secondary metabolism.

Grafting commercial grapevine varieties on interspecific rootstocks is a common cultural practice for improving stress resistance and vigor. Corso et al. reported on the acceleration of grape ripening under the influence of a new rootstock comparing to a commercial one. Molecular and biochemical analyses revealed that auxin signaling is strongly affected by the rootstock genotype and the existence of a link between the rate of berry development and the modulation of auxin metabolism which is more pronounced in skin tissue. On the other hand, Habran et al. addressed the combined effects of nitrogen supply and rootstock on berry composition. The authors used four rooststock/scion combinations fertilized with three different levels of nitrogen. The results showed complex responses of the metabolites' content (sugars, organic acids, aminoacids, anthocyanins, flavonols, flavan-3-ols/procyanidins, stilbenes, hydroxycinnamicB, and hydroxybenzoicacids) that depend on soil composition/rootstock/scion/climate interactions. These studies are fundamental in modern viticulture in order to clarify the impact of rootstock on berry scion development and ripening, and how this can be affected by adjustments in nitrogen fertilization.

The foliar exogenous application of kaolin, a radiation-reflecting inert mineral, has been proven effective in mitigating the negative impacts of abiotic stresses in grapevine. By performing a combined molecular and biochemical analysis, Conde et al. showed that kaolin treatment stimulated the expression of genes, and the activity of enzymes involved in the phenylpropanoid, flavonoid, and stilbenoid pathways at latter ripening stages. Metabolomic analysis corroborated this data and indicates that application of kaolin in grapevine leaves may be used as a summer stress mitigating strategy with positive impacts on berry quality.

Postharvest decay impacts significantly fruit quality and market value especially in climacteric fruits such as papayas that present a short-term shelf life. Liu et al. studied the role of *GH3* genes (encoding IAA-amido synthetases) in the regulation of postharvest physiology in papaya. The observed induced IAA-amido synthetase activities during the postharvest period may lead to the maintenance of low levels of endogenous IAA which is an inhibitor of ripening. Additionally, ascorbic acid treatment seems to regulate postharvest fruit ripening and softening by inducing a decrease in *GH3* genes expression, and IAA-amido synthetase activities, and therefore promoting endogenous IAA levels. Fruits treated with ascorbic acid showed a relatively lower production rate of ethylene than non-treated. These findings may provide a way to develop novel strategies for improving fruit quality during postharvest storage.

## Author contributions

AF wrote the editorial with input from AG, MP, and MB.

### Conflict of interest statement

The authors declare that the research was conducted in the absence of any commercial or financial relationships that could be construed as a potential conflict of interest. The reviewer GM and handling Editor declared their shared affiliation, and the handling Editor states that the process met the standards of a fair and objective review.
